# Green Labs: a guide to developing sustainable science in your organization

**DOI:** 10.1111/imcb.12624

**Published:** 2023-02-21

**Authors:** Joanne Durgan, Marta Rodríguez‐Martínez, Brendan Rouse

**Affiliations:** ^1^ Signalling ISP Babraham Institute Cambridge UK; ^2^ Genome Biology EMBL Heidelberg Heidelberg Germany; ^3^ Administration & Operations EMBL Heidelberg Heidelberg Germany

**Keywords:** climate change, environment, sustainability, sustainable science

## Abstract

Scientific research plays a vital role for society, but carries a significant environmental footprint, involving intensive use of energy and resources. Scientists are well placed to understand the unfolding climate and ecological crises, but may not appreciate how heavily their research, and other work‐related activities, contribute to emissions and pollution. With the consequences of climate change and ecological breakdown playing out in real time, scientists now have an important, urgent role to play in catalyzing solutions. Here, we explore how research organizations can reduce their environmental impact, share useful resources and encourage the global community to engage in making science more sustainable.

## Graphical abstract

Scientific research is vital for society, but bears a large environmental footprint. Here we discuss the role scientists can play in tackling the unfolding climate and ecological crises, and provide guidance on developing sustainable science in research organizations.

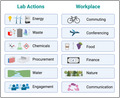

## INTRODUCTION

We are living through a perilous period in history, in which human impacts on the environment critically threaten our own well‐being and survival. Multiple, interrelated dangers, including climate change, pollution, habitat destruction and loss of biodiversity, seriously undermine the “safe operating space” in which human civilizations have developed and thrived.^1^, ^2^ Without urgent and ambitious action, these impacts are set to escalate, increasing risk of death and damage; political, economic and societal instability; and mass migration and conflict,^3^ posing a potentially existential threat.^4^

Climate change represents the single greatest challenge faced by humanity,^5^ driving extreme weather events, food shortages and water insecurity, and thereby threatening millions of lives around the world.^6^, ^7^ These impacts are experienced disproportionately in communities least responsible for anthropogenic emissions, and least able to fund adaptive measures, creating a major social injustice that exacerbates existing inequities.^7^ Despite decades of warnings, human‐induced emissions have continued to rise,^8^ and we are now at imminent risk of triggering cascading climate tipping points.^9^

The climate crisis also intersects critically with human health. Climate change undermines the basic determinants of survival, including food, water and shelter, and increases the risk of exposure to extreme heat and other dangerous weather events, including fires, floods, droughts and storms.^10^ Climate change also influences transmission of infectious diseases, favoring the spread of malaria, dengue, Zika and chikungunya by mosquitoes; cholera by *Vibrio cholerae* and a range of other water, air, food and vector‐borne diseases.^10^ Meanwhile, the air pollution associated with burning fossil fuels is estimated to kill millions of people/year worldwide,^11^, ^12^ with further links to lung cancer emerging.^13^ These combined impacts seriously undermine public health and sustainable development, and climate change represents the single greatest global health threat of the coming century.^14^

Urgent and ambitious action is required to address these unfolding global climate and ecological crises. After decades of delay, people around the world are already suffering the dire consequences of a warming climate.^7^ To limit further heating to well below 2°C, in line with the Paris Climate Agreement, modeling indicates we must halve our emissions each decade, reaching net zero by 2050.^15^ This window of opportunity is open, but closing rapidly, and the action we take right now will determine the scale and severity of future impacts. Action will be required at all levels—individual, organizational and governmental—to achieve the necessary transformative change.^16^, ^17^ Action within organizations lies in a productive, intermediate niche—scaling up significantly on individual impacts, but quicker and easier to implement than governmental change.

Against this backdrop, many scientific organizations are now working toward sustainability in their research.^18^ As scientists, we have a central role to play—we are well placed to understand the risks of climate change, pollution and biodiversity loss, and to develop solutions to these problems.^19^ However, there is perhaps an underappreciation of the environmental footprint of our research, which is incredibly energy and resource intensive.^20^ There is an inconsistency between undertaking research to benefit human health, but doing so in a manner that exacerbates the biggest threats to our environment and well‐being. “Green Labs” schemes seek to address this contradiction, promoting more sustainable approaches.

Given the multitude of equipment deployed in a typical research laboratory—from fridges, freezers, incubators, hoods and centrifuges, to microscopes, PCR and FACS (fluorescence‐activated cell sorting) machines—and the associated requirements for heating, cooling, ventilation and lighting in the buildings that house them, it is staggering, but perhaps not surprising, that laboratories consume 5–10, sometimes up to 100, times the amount of energy used by an equivalently sized office or commercial space.^21^ Life science research also consumes a huge volume of plasticware, estimated at roughly 5.5 million tons per year,^22^ which would account for nearly 1%–2% of global plastic use.^23^ Further impacts are associated with consumables, chemicals, materials and equipment, massively amplified *via* associated supply chains.^24^, ^25^ The carbon output of biotech and pharmaceutical companies alone is nearly 200 million tCO_2_e, roughly equivalent to half of UK emissions (and not including academic laboratories).^26^ While these figures are shocking, they do reveal many opportunities for practical actions that can directly reduce emissions and pollution at scale. Many of these actions will also save money in the long term, which can be reinvested back into research.^27^

**Figure 1:**
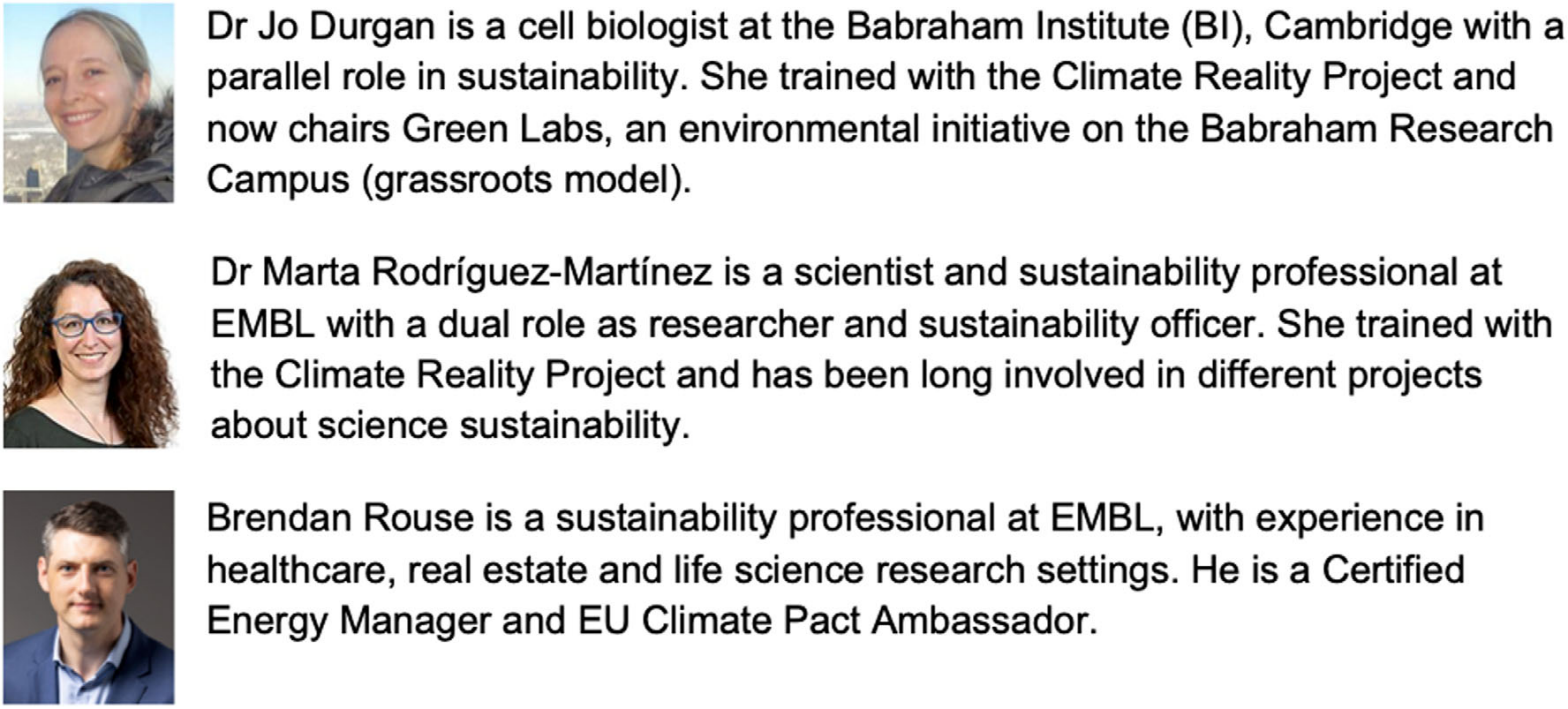
About the Authors. Background information on each of the article's authors.

Here we share ideas and insights on approaching environmental responsibility in a research setting. We authors have come to work on sustainable science *via* different routes (Figure 1): either as scientists who felt compelled to take environmental action (Jo Durgan – JD and Marta Rodríguez‐Martínez – MR‐M), or as sustainability professionals choosing to work in a scientific setting (Brendan Rouse – BR). Through these mirrored perspectives, and from within different organizations, we aim to provide some complementary insights, to learn from each other and to share experiences and resources with anyone who recognizes the urgency of taking action and decides to start a Green Labs initiative in their own setting.

## DECIDING TO ACT

As we all become increasingly aware of the unfolding climate and ecological crises, many of us will reach a point where we feel compelled to act. Fortunately, a wide range of solutions are already at hand,^28^, ^29^ and there are many possible ways to contribute.

**Figure 2:**
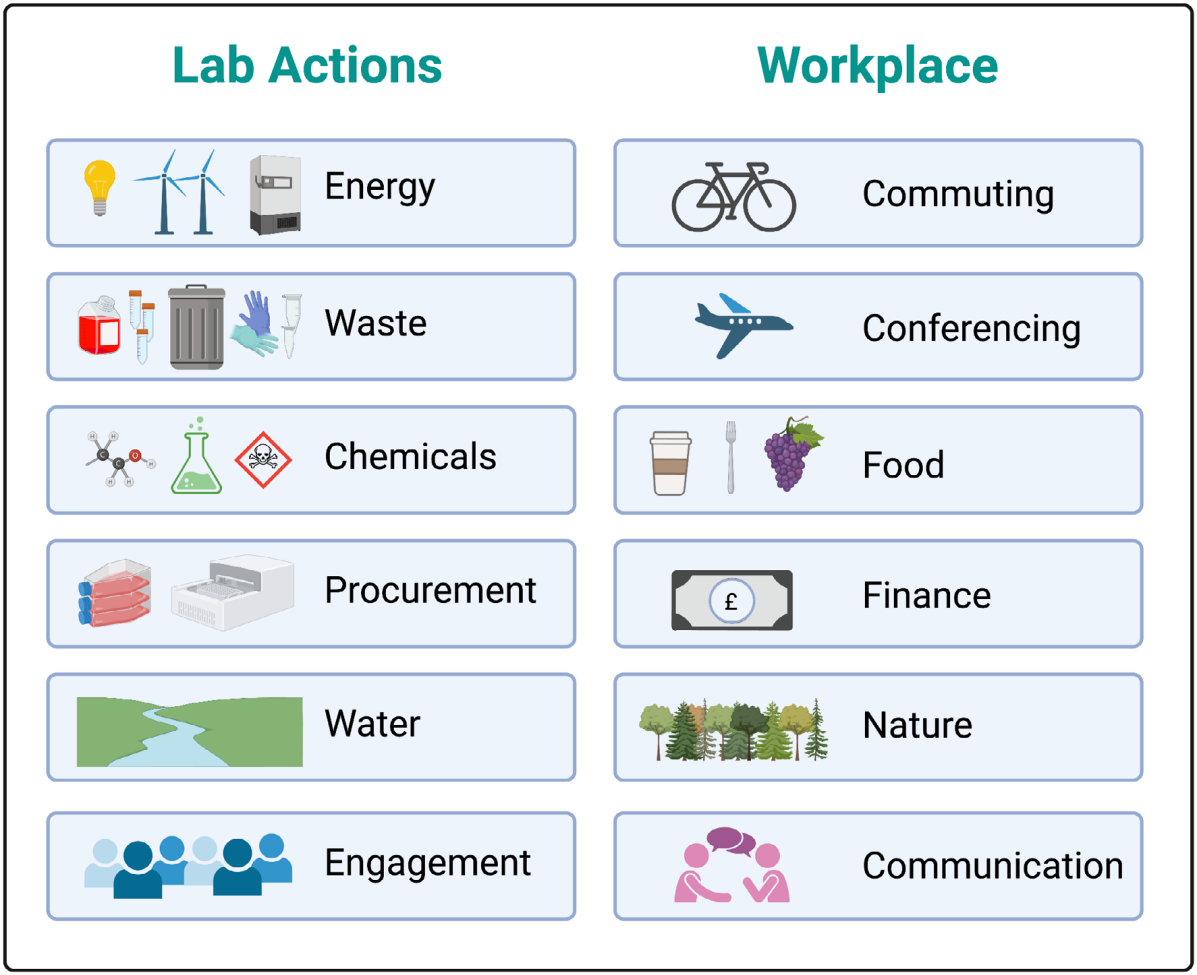
Key areas for sustainability action in research laboratories and across the wider workplace. This figure was created using BioRender.com.

JD: It's worthwhile reflecting at the outset on what approach to environmentalism might suit you best—the “Climate Venn” is a great concept for matching individual skills and interests with climate actions.^30^ For those of us based in laboratories, the workplace can be a highly productive niche. On a practical level, actions can be focused on energy, waste and water in laboratories; on transport, food and finance in the wider workplace and on broader communications, engagement and projects centered on community or nature (Figure 2). From a grassroots perspective, a major benefit is that identifying and implementing possible measures is much easier in a familiar setting, which you already largely understand. You also have existing relationships with colleagues, and an established network, to work within. These benefits can get your activism off to a running start.

BR: Once an organization takes the decision to act, this can be delivered by giving recognition and resources to grassroots initiatives, by modifying existing job roles to fulfill this role or through the creation of a dedicated department that takes responsibility for the transition to sustainability.

## BECOME INFORMED

**Figure 3:**
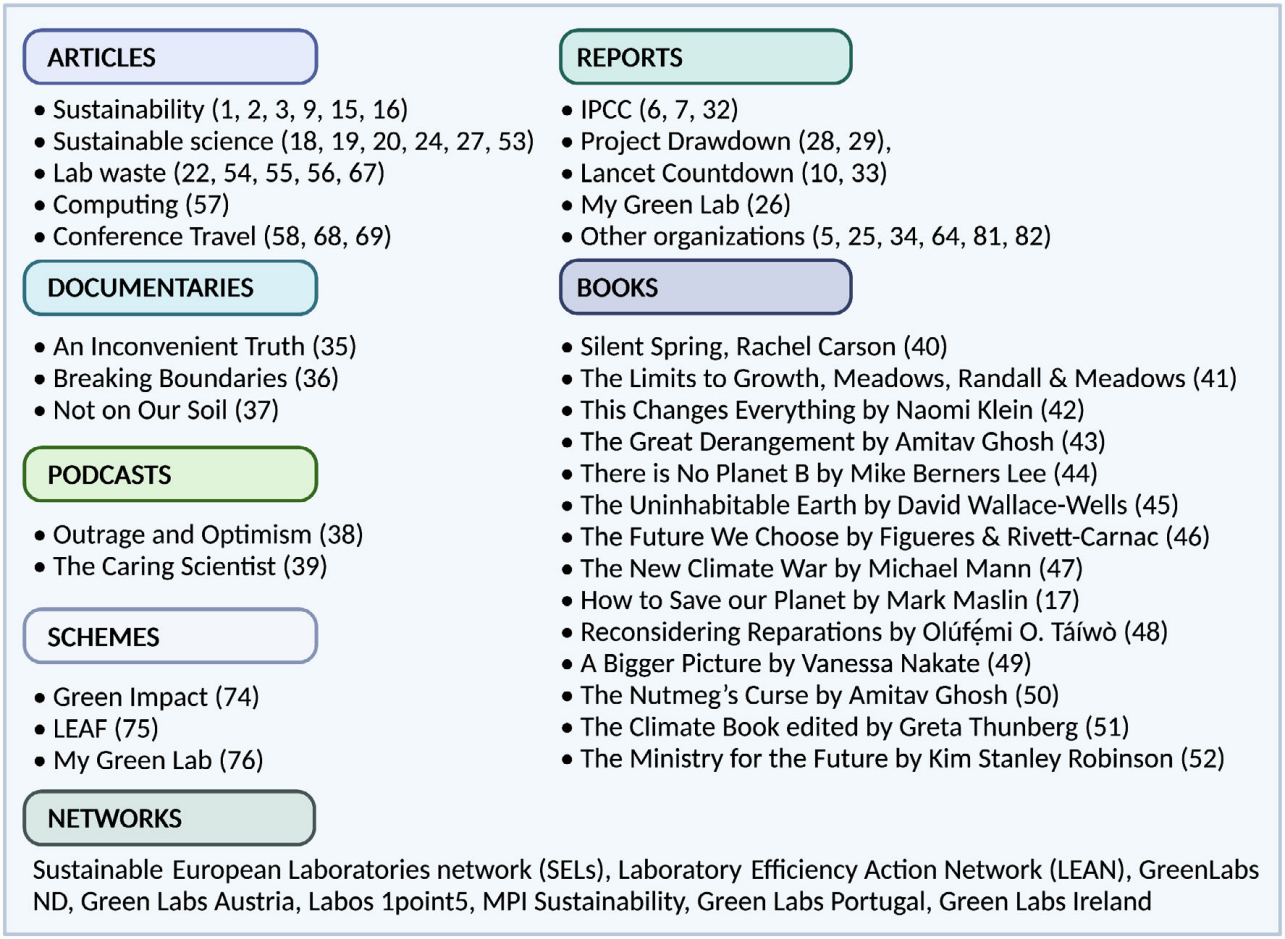
A selection of the many resources available to learn about environmental issues and sustainable science ^1^, ^2^, ^3^ ^,^ ^5^, ^6^, ^7^ ^,^ ^9^, ^10^ ^,^ ^15^, ^16^, ^17^, ^18^, ^19^, ^20^ ^,^ ^22^ ^,^ ^24^, ^25^, ^26^, ^27^, ^28^, ^29^ ^,^ ^32^, ^33^, ^34^, ^35^, ^36^, ^37^, ^38^, ^39^, ^40^, ^41^, ^42^, ^43^, ^44^, ^45^, ^46^, ^47^, ^48^, ^49^, ^50^, ^51^, ^52^, ^53^, ^54^, ^55^, ^56^, ^57^, ^58^, ^59^ ^,^ ^64^ ^,^ ^67^, ^68^, ^69^ ^,^ ^74^, ^75^, ^76^ ^,^ ^81^, ^82^. This figure was created using BioRender.com.

For a biomedical bench scientist, transitioning into sustainability can feel daunting, encompassing a wide range of areas which fall outside professional expertise. Fortunately, a wealth of information and resources are available, covering the environmental challenges we face, their solutions and how to embed sustainability into our science (Figure 3). Exploring these resources and becoming well‐informed is an important early step.

JD: A huge body of climate change literature dates back at least as far as the pioneering work of Eunice Newton Foote,^31^ and a succession of scientists have developed our understanding and warned us clearly of the risks associated with climate and ecological breakdown. While this list is by no means exhaustive, some great resources include (1) reports,^25^, ^28^, ^32^, ^33^, ^34^; (2) documentaries^35^, ^36^, ^37^; (3) podcasts^38^, ^39^; (4) books^40^, ^41^, ^42^, ^43^, ^44^, ^45^, ^46^, ^47^, ^48^, ^49^, ^50^, ^51^ and even thought‐provoking fictional works.^52^ A range of excellent courses and training opportunities are also available, personally I have benefited from: Climate Reality Leadership, (www.climaterealityproject.org/training), Planetary Boundaries (www.edx.org) and Zero Carbon Britain (https://cat.org.uk) training. A growing collection of resources is also available to learn more about sustainability in a scientific setting, including reports^26^ and articles on sustainable science^18^, ^19^, ^20^, ^24^, ^27^, ^53^ waste in laboratories,^54^, ^55^, ^56^ greener computing^57^ and sustainable conferencing.^58^ Many of these issues are also covered on The Caring Scientist: Mission Sustainable podcast.^39^

MR‐M: Many additional resources are available online from organizations (e.g. My Green Lab), programs [e.g. Laboratory Efficiency Assessment Framework (LEAF), Green Impact], groups and networks (e.g. SELs, LEAN, GreenLabs ND, Green Labs Austria, Labos 1point5, MPI Sustainability, Green Labs Portugal, Green Labs Ireland).

BR: It is also important that anyone who wants to drive sustainability at work understands the way their organization functions and therefore how to facilitate change. Smaller research centers may not work the same way as universities or larger facilities, and similarly, different departments within the same organization can have individual approval processes and ways of working. Understanding how your organization makes decisions, the personalities and culture in different departments and what drives them makes it easier to effectively shape arguments and deliver sustainable change.

## BUT DO NOT WAIT TO BE AN EXPERT

As researchers, we are accustomed to mastering our field, and the associated literature, in depth and detail. However, it is not necessary to become an expert in sustainability before taking some action; in fact, that approach may slow you down. Many impactful actions have been well‐established by others (see the “Involve Senior Staff” section), which can be replicated in your setting, to kick‐start a green transition.

JD: Achieving some quick wins can build momentum and shift culture, while you continue to learn. At BI (Babraham Institute), we focused first on energy efficiency by racking and “chilling up” our **−**80°C freezers to **−**70°C. Each unit uses roughly the same amount of energy as 1–2 average households,^59^ and we have a suite of about 40. Making this shift reduced their energy consumption by nearly 20%, and extends freezer lifetime.^60^ This action was targeted, achievable and impactful, creating a foundation from which to develop our work.

MR‐M: A lot of the actions and initiatives that start from grassroot movements can subsequently scale‐up and include additional departments, such as communications, facilities management and finance. By collaborating with specialists in these areas, one can bypass the need to become an expert in all domains regarding research sustainability.

BR: It is also important to recognize the actions already being taken by colleagues whose expertise make the organization more sustainable (even if they did not realize it themselves). Take the laboratory kitchens at EMBL Heidelberg, who did not appreciate they save 350 plastic bottles a week by preparing sterile reagents. Or, our Scientific Instrument Maintenance team, who created a circular process for laboratory equipment through fixing and salvaging equipment which would otherwise be discarded and replaced with new. We have promoted these teams and shown how vital they are to EMBL's sustainability ambitions. We have found that after we speak to them, and show how they already contribute to our work, they become even more engaged, and we can leverage their expertise better. In fact, the two teams mentioned here have since initiated new recycling and energy‐saving initiatives, respectively.

## BUILD A TEAM AND ORGANIZE

An effective next step is to build a team within your organization, who can share ideas and expertise and help plan and implement a wider set of actions.

JD: At BI, we established a Green Labs Steering Group, composed of volunteers from across our organization, with a common interest in the environment. In this way, we can draw on the knowledge of engineers, waste managers, estates, operations, health and safety and those working in administration, finance and communications, as well as scientists in a range of roles, including students, postdocs, research assistants, facility heads and group leaders. In building a team, we have placed a strong emphasis on equality, diversity and inclusion, striving for well‐balanced representation within our team, and linking up with environmentalists representing different groups and regions.^61^, ^62^

While shared enthusiasm got us started, we have found value in adopting more organized arrangements as our work has evolved, and now gather for quarterly meetings, with a structured agenda, minutes and action points. This more formal approach has helped us organize and catalyze actions, navigate decision making more readily and interface with senior staff and governance structures more effectively, to share recommendations and influence policy.

MR‐M: At EMBL, an active grassroots group is long established, run from the EMBL Staff Association. This group has delivered events and highlights issues important to their members. We also have green groups on our sites, such as the EMBL‐EBI green network, which work closely with the host organization to raise issues. In Barcelona, our team sits on the sustainability network of the host organization.

At the organizational level, and because EMBL has an Environmental Office, sustainability is interwoven in existing governance structures, meaning it is discussed in many meetings which bring together all operational leads, or specialist groups looking at specific topics, such as the current energy crisis. Furthermore, sustainability items are regularly discussed within the highest governance levels of the organization. This has the benefit of allowing projects to be delivered quickly once they are approved.

## INVOLVE SENIOR STAFF

Securing the involvement and support of senior staff is hugely beneficial to a Green Labs initiative, and sends a strong signal on the importance of sustainability within an organization. On a practical level, this engagement may be required to secure investment and/or personnel for certain actions. Feed‐in and advice from experienced colleagues can also bring a valuable, alternative perspective, along with the power to change policy.

BR: There are many areas where staff want clear policies and guidelines within which to operate and this needs leadership involvement. It can take time, requiring a lot of discussion and consultation. EMBL's sustainability team regularly engage with the senior leadership. For example, we recently piloted a sustainability scheme (LEAF) with 13 research groups, the results of which were shared with the organization's highest level of operational decision makers, the Directorate. We demonstrated the majority of participating groups found the assessment to be user‐friendly and relevant. Following this, the Directorate gave its approval for LEAF to be rolled out to all of EMBL's groups, with the organizational support needed to make it a success.

JD: At BI, we have also benefited from the engagement of senior staff. BI's Director is our project sponsor, our Green Labs group shares members with the Babraham Executive Committee and other senior colleagues share ideas and encouragement, driven by personal concern over the environment. We also submit formal, periodic reports to senior management, ensuring our environmental agenda is raised, discussed, minuted and acted upon at regular, high‐level meetings. Together, these inputs help create a culture of sustainability at all levels, in which Green Labs action can thrive.

If the prevailing culture or leadership of your organization is less engaged, it may help to highlight the sustainability work underway at similar institutions, or invite an external speaker to present. Peer‐to‐peer learning and comparisons help shift perceptions, reset expectations and highlight the many cobenefits of taking action. We have delivered talks at several universities and institutions across the UK/Europe to encourage wider action, and are happy to provide this presentation to others. The growing network of scientists engaged in sustainability offers a wide range of other possible contacts or speakers.

## PLAN YOUR ACTIONS

The broad areas encompassed by a typical Green Labs initiative are summarized in Figure 2, spanning both research and other work‐related activities. Within laboratories, important areas include (1) energy (efficiency, equipment switch off, computing), (2) waste (reducing, reusing and recycling of consumables, chemicals and organic waste), (3) water (reducing consumption, responsible disposal of contaminants), (4) procurement (choosing responsible suppliers, addressing the impact of supply chains) and (5) engagement (building a culture of informed and active colleagues, sharing knowledge with other scientists and the wider community).

JD: To develop our Green Labs program, we combined preexisting good practice with design and implementation of new actions, seeking to target each of the key areas shown in Figure 2. A wide range of possible actions can be considered: for detailed guidance please see the references and recommended resources (Figure 3). Some actions will be applicable across most laboratories (e.g. switching off equipment when not in use), while others may vary according to setting and/or budget (e.g. ventilation and lighting). At BI, our overarching criteria are that new actions must not disrupt research quality or compromise on health and safety.

While planning our actions, we have drawn on the collective expertise of our team, online resources and advice from our network, and encourage all staff members to feed‐in recommendations—people know their own work areas best and often identify specific actions we had not thought of. We aim to think quantitatively, ideally identifying and prioritizing higher impact areas first. However, a major obstacle we face here is that our grassroots team lacks the capacity to rigorously monitor and quantify. Instead, we take guidance on likely impacts from the work of others (e.g. My Green Lab) and measure where we can (e.g. using simple energy monitors, then extrapolating estimated savings). Other barriers to action for us include (1) time constraints, particularly as a grassroots team developing Green Labs alongside our primary roles; (2) costs, where up‐front investment is required and (3) logistics, for instance, where a new waste stream requires additional space, bins or a modified collection protocol. We have overcome, or worked around, some of these obstacles and find that each new action helps build momentum for the next. However, our Green Labs project remains an ongoing and evolving project with much work left to do.

BR: At EMBL, we used a well‐established methodology to identify the most significant environmental issues for the organization called a Materiality Assessment.^63^ This required engagement with internal and external stakeholders, to discuss environmental impacts, followed by ranking of different environmental/sustainability issues, based on the importance our stakeholders place upon them, and the impact they have on the organization. Our Assessment also ranked EMBL's environmental impact with regard to each of the issues. We spoke to members of staff at all levels, across a range of roles, and to major funders, other research organizations and local authorities, to get a broad range of views on our environmental impacts. The results of the assessment gave EMBL 10 topics to focus on which now make up the organization's sustainability strategy,^64^ organized into three distinct workstreams: (1) doing environmentally responsible research; (2) doing environmentally relevant research and (3) promoting sustainable science.

It is important that where possible, specific targets are set to ensure the strategy is ambitious and its progress can be monitored. EMBL has set targets for energy‐based carbon footprint (50% reduction), business travel (50% reduction), waste generation (30% reduction) and recycling rate (90%), all by 2030 (against the 2019 baseline), as well as eliminating non‐essential single‐use plastic by 2025. To create action plans to achieve these targets, specialist working groups were set up which developed short‐, medium‐ and long‐term actions, as well as key performance indicators for the first year.

## TAKE YOUR FIRST ACTIONS

Each research organization is unique, so will need their own approach to planning and implementing environmental actions. In general, anyone interested in sustainable science should familiarize themselves with their workplace and explore what they can control and where they can take their first actions. Some examples from BI and EMBL are as follows.

### Energy

JD: On a site level, BI use a Trigeneration system to combine heating, cooling and power, saving nearly 2500 tCO_2_ emissions and > £1 million over the last 3 years. On a laboratory level, we have retired an obsolete 37°C room and are shifting **−**80°C freezers to **−**70°C. We are now focusing on broader energy saving actions in all laboratories, such as more diligent switching off of equipment, lights and computers when not in use. Each such action may be smaller, but together they have a large cumulative impact across many laboratories and through time.

BR: At EMBL, we have installed a solar array to provide renewable energy and we are making changes to operating conditions, including setting heating to a maximum of 19°C, no longer heating unoccupied spaces such as stairwells and corridors and removing hot water to handwashing sinks. We are increasing the temperature in our data centers from 20°C to 24°C, and migrating data services to more efficient centers. Our laboratories are being provided with multiplugs and switches, to make it easier for users to turn equipment off, and we are encouraging energy‐saving behavior through posters, stickers and communication channels.

### Water

JD: At BI, retrofitting of heavily used autoclaves, with a system that uses recirculating chilled water, has saved about 32 000 L of water per week.

### Waste

JD: We benefit from established good practice on waste management at BI, and aim to build on this further, applying a hierarchy of reduce, reuse, repair THEN recycle: We (1) *reduce* shipments and packaging waste by consolidating orders from across the Institute, and running a ‘Central Stores’, where commonly used items are stored in bulk and can be purchased on site; (2) *reuse* lab equipment and furniture, internally and *via* the UniGreen scheme; (3) *repair* laboratory equipment where appropriate, though this must be counterbalanced against possible efficiency improvements in newer kit and (4) *recycle* a wide range of materials, including paper, glass, batteries, metal, wood, some plastics, printer cartridges and e‐waste, as well as organic waste by composting; the remainder is sent to an energy‐from‐waste facility to avoid landfill. Developing new waste streams can be complicated, particularly for a grassroots team, involving stringent health and safety requirements and logistics. As such, we have focused strategically on one commonly used item at a time, for example, establishing a new recycling stream for plastic media bottles, which diverts about 45 000 bottles/year from incineration.

BR: At EMBL, we are also working to improve our waste management systems and have some good practices established. It is worth noting that we operate in five different European countries, with different host organizations/landlords, so waste management is different at each site, performed in the context of local provisions. For example, we use our local authority waste services in Heidelberg (Germany) to recycle packaging waste, but they do not have a service for consumables, so we use a local company to collect and recycle tip boxes and bottles. Meanwhile, in Hinxton (UK), our host organization uses a private company to provide a full collection and recycling service. Understanding the local waste management ecosystem is vital to ensure waste is treated as sustainably as possible.

MR‐M: Working up the waste hierarchy, EMBL have some great examples of local initiatives. Our Grenoble (France) site collects chipped and cracked glassware and sends it to a local company to be fixed, rather than replaced. In Heidelberg (Germany), our Scientific Instrument Maintenance team repairs an array of electronic and mechanical equipment. In Barcelona (Spain), the local team arranges waste quizzes and events to promote more sustainable waste management practices.

ALL: These examples illustrate that reuse and recycling are feasible in a scientific setting, but that responsible waste management is heavily dependent on appropriate services and infrastructure, which may not yet be available in all countries or regions. In this case, alternative focus might be given to advocating locally for improved recycling provisions or manufacturer take‐back schemes, and/or on reusing or substituting unsustainable products.

### Consumables

JD: The environmental impact of consumables and supply chains is enormous.^20^, ^26^ BI belongs to a consortium which has developed a Responsible Procurement Strategy,^65^ addressing environmental, social and ethical responsibility and aligning with the UN Sustainable Development Goals. We also engage directly with suppliers to reduce packaging and shipments and invited a guest writer to blog on reuse of labware.^66^ Notably, a team of BI scientists have developed methods to reduce antibody consumption for FACS by about 10‐fold,^67^ which will make significant savings, over time and across multiple laboratories, all the way up the associated supply chain.

## THINK OUTSIDE THE LAB TOO

Our jobs involve more than just our research and we can also consider the environmental impacts, and opportunities, associated with broader work‐related activities too (Figure 2), from commuting and conference travel to food and finance, as well as engagement with our wider community and care for our local surroundings.

JD: Transport is a particular issue for BI, which sits a few miles outside Cambridge. To address the impact of staff commuting, we offer a bus pass, providing free travel for students and subsidized journeys for other staff, and benefit from a great network of cycle paths, bike sheds and related facilities (e.g. showers, lockers). However, to reduce reliance on single occupancy, fossil‐fuel vehicles there is still much more to do. Moving forward, we are keen to provide additional cycling benefits, facilitate ride‐sharing and increase electric vehicle use (ebikes and electric cars).

The collective impact of conference travel is another important area for scientists to consider.^58^, ^68^ We are raising awareness through communications, asking staff to reflect on when and why air travel is required, factoring in the value of a specific event, as well as career and project stage, to prioritize only the most important conferences for flights, while supplementing with additional virtual or local events.^69^ We encourage staff to take alternative transport wherever possible, strongly discouraging domestic flights, advocating for rail travel where reasonable (e.g. Eurostar) and sharing useful resources on journey planning^70^; a major barrier here is the added cost of rail travel compared with cheap flights, as well as time burden over longer distances. Finally, while carbon offsetting is a contentious issue,^71^ BI have decided to offset unavoidable flights by supporting Verified Carbon Standard projects with social cobenefits, such as carbon avoidance (e.g. cookstove replacement) and renewable energy generation.

Within our workplace, there are other areas where we lack direct control, yet can still advocate for sustainable change. For example, we engaged with our caterers to promote plant‐rich, local and seasonal menu options and to discourage single‐use items and food waste. With respect to finance, we have advocated for fossil‐fuel divestment, drawing parallels with tobacco, which is already classed as an unethical investment. Next, we aim to provide information on pensions to interested staff, as choosing an ethical pension provider is one of the most impactful actions an individual can take.^72^

Finally, we have supported actions on nature and biodiversity on our campus, which is housed on a ~400‐acre estate comprising a range of habitats, including meadows, woodland and a chalk stream. We have run activities with the local school, planting 1000 native hedges and collecting litter, and supported the creation of a new Forest Garden with Babraham Village residents. These active, nature‐based projects play an outsized role in fostering a culture of participation and positivity, supporting team building and creating a visible movement of environmental responsibility.

BR: We must also consider the environmental impact of new buildings as nearly 40% of global emissions are attributable to buildings and construction.^73^ The impact of a new laboratory building must be considered during its design, construction, while it is in operation and finally when it is to be demolished. Because of the energy‐intensive operation of laboratories, and the structural nature of the buildings which need to support heavy equipment, or limit vibrations for sensitive measuring and imaging, it is not uncommon for laboratories to use four times as much energy as a commercial office building, and have two times as much embodied carbon.

## JOIN A SUSTAINABILITY SCHEME

Another helpful step to consider, particularly as your sustainability efforts grow, is joining a formal scheme to help guide, organize and reward your work—examples include Green Impact,^74^ LEAF^75^ and My Green Lab.^76^ These schemes provide a framework of established actions, to help plan and structure work. Having a deadline to aim for, and certain award levels to target, can also catalyze action, and provide an external motivator when requesting support. It can also be rewarding for team members to have their efforts recognized with accreditation, and provides the reputational benefits of receiving an award for your workplace.

BR: At EMBL we are rolling out LEAF following a successful trial. During the pilot, we identified where we needed to provide additional support for the participating groups, which led to the introduction of a new recycling waste stream. We have also created guidance and resources for the participating groups. This support, and our groups’ enthusiasm, resulted in all 12 of the pilot groups achieving the criteria for a bronze rating.

JD: At BI, we just completed our first year of Green Impact (SOS‐UK). As a grassroots team, the structure provided was really helpful, and we were delighted to receive a gold award. Another major benefit was the opportunity our participation created to recruit biotech companies to join us from across our shared Babraham Research Campus. Working together with SOS‐UK, we developed a bespoke online toolkit, shared among 10 separate campus organizations, who then used this resource as a “menu” of sustainability actions to select from, however suited them best. Taking this joint approach, we achieved over 500 environmental actions collectively last year, significantly amplifying the impact our institute could have made alone, and building a new and active sustainability network across our site.

## DEVELOP A WIDER NETWORK

A growing number of institutes, university laboratories and biotech companies are now working on sustainability in science and organizing to form co‐operative networks (Figure 3). Engaging with a wider network facilitates information sharing—to exchange advice, ideas and resources—and avoids duplication of effort. We can also learn from colleagues who have long‐applied efficiency measures, such as conserving energy and resources, owing to regional or institutional limitations on funding and resources.^77^

MR‐M: At EMBL we have developed collaborations with other research institutes in our locations (e.g. Heidelberg Sustainability Labs forum), on the campuses we share with other organizations (e.g. PRBB campus in Barcelona and the Wellcome Genome Campus) or with other similar intergovernmental organizations (e.g. EIRO Forum). The local groups provide a space for our staff to engage with peers and influence the decisions being made at their workplace. We are also able to benchmark our actions and strategy with other like‐minded organizations and share best practice.

JD: At BI, we have developed new relationships based on shared location (e.g. campus companies, local university laboratories), funding sources (e.g. other Biotechnology and Biological Sciences Research Council‐funded institutes (BBSRC)), membership of existing alliances (e.g. EU‐LIFE), personnel (e.g. when staff move to a new workplace) or simply through a shared interest in sustainable science (e.g. EMBL). We also engage more widely with environmental organizations (e.g. the Climate Reality Project) and initiatives (e.g. the Frontiers Planet Prize), and attend broader sustainability events (e.g. Centre for Alternative Technology, Aberystwyth; Global Systems Institute, Exeter). Building or joining a network, locally, nationally and/or internationally, helps us all move further with our work faster, and contributes to the collective action required to underpin transformative sustainable change.^16^

## COMMUNICATE EFFECTIVELY

For many, the climate and ecological emergencies already represent clear, major threats, demanding of urgent action. However, for others, these issues are not yet perceived as a high priority. By engaging in effective communication, both internally and externally, we can help build awareness and catalyze action within the scientific community and beyond.

JD: Working on a Green Labs initiative involves different types of communication, from technical information sharing to advocacy and engagement. Environmental issues such as climate change and biodiversity loss can be highly emotive, and discussing them involves acknowledging some incredibly sobering facts. For some people, fear may be an effective motivator for action, for others, an emphasis on optimism and solutions is important, to inspire action rather than despair; in all cases, clear, honest and well‐evidenced communication is critical.^78^

Encountering skepticism, or resistance to action, can feel frustrating, but in these situations, it is important to focus on your objective—is it to win the argument, or to achieve a particular change? Motivated by the latter, it can be helpful to focus on positive dialogs, identifying shared values (e.g. family) or common interests (e.g. nature). If the environment proves a polarizing topic, you can even sidestep it completely to focus on the cobenefits of sustainability (e.g. health benefits),

On a procedural level, you may at some point need to advocate for a specific action or policy change in your workplace. In our experience at BI, a good initial approach is to identify the relevant formal mechanism, and submit a clear, evidence‐based case for your request. You may find you are pushing at an open door, and that sharing a well‐reasoned argument to the appropriate decision makers is all it takes. If more persuasion is needed, try to understand, and then address, any perceived barriers, or appeal to other motivators (e.g. cost savings). Where possible, constructive dialogs and positive relationships are likely to be a good investment in the long term, especially given you may well encounter the same decision makers again in relation to future requests or actions.

BR: It is easy to feel frustration with colleagues and peers who do not seem to act in a sustainable way. However, we must recognize that most people act logically, fulfilling their motivations and acting within the barriers that are placed in their way. The reality is that climate change, biodiversity loss and the many other catastrophes we face can feel remote or impersonal and people often act in a way that responds to more immediate and direct needs. We have to take the time to listen and empathize with others to understand what their barriers and motivators are if we want to communicate effectively and with the goal of changing behaviors.

## AMPLIFY YOUR IMPACT

Working to address environmental problems, either as an individual or in your workplace, has an immediate, local impact, but also contributes momentum toward larger, social changes. By combining effective communication with wider engagement, it is possible to amplify your impact even further. Within the scientific community, BI and EMBL have presented talks, and participated in workshops, panel discussions and other events to raise awareness of environmental issues and sustainability in science. We have also encouraged other organizations to initiate their own sustainable science actions.

JD: At BI, we share information and inspiration, with our staff and others, in a variety of formats. Within our workplace, we provide regular content *via* our Institute newsletter, intranet pages, internal meetings, social media (@GreenBabraham) and blog posts, to foster a visible and consistent culture of sustainability. We include information on laboratory‐based actions (e.g. reducing plastic use),^66^ as well as stories of staff undertaking green projects in their personal lives (e.g. installing solar or switching to an electric vehicle).^79^ This approach generates a significant ripple effect—a recent blog on eco‐retrofit measures has inspired nearly 20 staff members to take new actions in their own homes.^80^ We also seek to share the platforms available to us, for instance, hosting guest blogs from other regions.^62^ Within our wider community, we have supported practical actions, such as litter picking and tree planting, and given talks to schools and community groups; while we are not environmental specialists, our scientific background lends some weight to our advocacy. Members of our team also support other international environmental projects (e.g. mentoring for the Climate Reality Project).

BR: After carrying out our Materiality Assessment, we realized that alongside doing environmentally responsible science, the biggest impact we can make is through the research we perform. Our sustainability strategy therefore has a section on “Environmentally Relevant Research” exploring the role molecular biology can play in solving some of the planet's biggest threats, and how we can develop links with the environmental community. One key action we have taken is attending COP26 (26th UN Climate Change Conference of the Parties), to promote the life sciences as a key tool in the fight against climate change.^81^ We also launched a new research theme at EMBL called “Planetary Biology” to understand, from the molecular to the population level, how microbes, plants and animals respond to each other and to their environment. Planetary Biology will address fundamental and pressing scientific questions about the influence of environmental parameters on the molecular mechanisms underlying biological processes, while also addressing societal questions about planetary health.

## SUMMARY

We are the first generation to fully understand the consequences of climate and ecological breakdown, and the last who still have the opportunity to limit the most extreme outcomes. No one can tackle these problems alone, but we can all be part of the solution. Most of the tools we need are already at hand, but we must choose to use them.

Green Labs initiatives, and organizations taking a stance to reduce their environmental impact, are great ways for the scientific community to take direct, practical action and play their part in driving transformative change. However, action by individual researchers and organizations will not be enough. To build further momentum, we will need engagement from all those bodies who can influence even larger groups. We suggest that:

1. Funders should expect (and then insist) that their funds be spent efficiently and with environmental responsibility, acknowledging they bear some accountability for the impact of the research they support.
2. Suppliers must manufacture products that are more sustainable, across their full life cycle and whole supply chain, and make these products more attractive than their alternatives.
3. Conference organizers should offer virtual participation and/or local hubs to reduce travel impacts (also yielding benefits with respect to equity and inclusion).
4. Publishers have a responsibility to raise awareness on all of the above.

Encouragingly, this type of engagement is clearly growing, through the development of sustainability strategies,^25^, ^64^, ^82^ advocacy movements (e.g. https://www.sustainablescienceadvocates.org) and an increasing number of papers published on the environmental impact of research (see References and Figure 3).

We now encourage our colleagues, around the world, in all science‐related roles and at all levels, to join in these collective efforts to make our science more sustainable and help affect lasting, transformative change.

## AUTHOR CONTRIBUTIONS

**Joanne Durgan:** Conceptualization; visualization; writing – original draft; writing – review and editing. **Marta Rodríguez‐Martínez:** Writing – original draft. **Brendan Rouse:** Writing – original draft; writing – review and editing.

## CONFLICT OF INTEREST

The authors declare they have no conflict of interest.
